# Enhanced Spectral Response of ZnO-Nanorod-Array-Based Ultraviolet Photodetectors by Alloying Non-Isovalent Cu–O with CuAlO_2_ P-Type Layer

**DOI:** 10.3390/nano13091472

**Published:** 2023-04-26

**Authors:** Yuchen Long, Ziling Zhang, Xiutao Yang, Yang Liu, Guangcan Luo, Jingquan Zhang, Wei Li

**Affiliations:** 1 College of Materials Science and Engineering, Sichuan University, Chengdu 610064, China; 2 School of Materials Science and Engineering, Guizhou Minzu University, Guiyang 550025, China

**Keywords:** ZnO nanorod arrays, UV photodetector, spectral response

## Abstract

CuAlO_2_ was synthesized by a hydrothermal method, in which the Cu–O dimers were incorporated by simply altering the ratio of the reactants and the temperature. The incorporation process increases the grain size in CuAlO_2_, and modulates the work function and binding energies for CuAlO_2_ due to the partial substitution of Cu^+^ 3d^10^ with Cu^2+^ 3d^9^ orbitals in the valence band maximum by alloying non-isovalent Cu–O with a CuAlO_2_ host. Based on the ZnO nanorod arrays (NRs) ultraviolet photodetector, CuAlO_2_/Cu–O fabricated by the low-cost drop-coating method was used as the p-type hole transport layer. The incorporation of the Cu–O clusters into CuAlO_2_ lattice to enhance the conductivity of CuAlO_2_ is an effective way for improving ZnO NRs/CuAlO_2_ device performance. The photodetectors exhibit significant diode behavior, with a rectification ratio approaching 30 at ±1 V, and a dark saturation current density 0.81 mA cm^−2^. The responsivity of the ZnO-NRs-based UV photodetector increases from 13.2 to 91.3 mA/W at 0 V bias, with an increase in the detectivity from 2.35 × 10^10^ to 1.71 × 10^11^ Jones. Furthermore, the ZnO NRs/[CuAlO_2_/Cu–O] photodetector exhibits a maximum responsivity of 5002 mA/W at 1.5 V bias under 375 nm UV illumination.

## 1. Introduction

Ultraviolet (UV) photodetectors have come to be used effectively in numerous fields, such as detection of missiles, solar UV monitoring, and ozone hole detection [1]. In the process of detector development, one-dimensional (1D) nanomaterials have attracted considerable attention, owing to the excellent photoelectric properties of their ultrahigh intrinsic photoelectric, quantum confinement effect, multiple light confinement, and subwavelength size effects [2]. Due to the large surface-to-volume ratio and 1D nanostructure, the 1D nanomaterial can serve as a carrier transport channel. Thus, the transport time can be shortened and the lifetime of photogenerated holes and electrons can be extended, which leads to high response and internal photoconductive gain [2,3]. For example, SnO_2_- and ZnO-nanowire-based devices exhibit higher transport rate than their nanoparticle-based counterparts [4]. Therefore, some 1D nanomaterials, such as SiC, NiO, ZnSe, AlGaN, and ZnO, have become commercially available [5]. Among them, ZnO is one of the essential wide-bandgap semiconductors, with a direct bandgap of 3.37 eV and excitation binding energy of 60 meV at room temperature, as well as a non-centrosymmetric crystal structure [6]. On account of its unique properties such as environmental friendliness, abundant raw materials, and chemical stability, ZnO has been considered as a promising photoelectronic material [7]. Until now, it has been synthesized by a wide variety of chemical and physical techniques, such as chemical vapor deposition [8], pulsed laser deposition [9], molecular beam epitaxy [10], and the hydrothermal method [11]. Due to the low cost, controlled shape, and large-area compatibility, the hydrothermal method is extremely suitable for the fabrication of high-quality ZnO NRs [12,13]. In terms of applications, ZnO NRs have, until now, been integrated into light-emitting diodes (LEDs) [14], thin-film transistors [15], and gas ionization sensors [16].

Several typical types of 1D ZnO-NRs-based self-powered photodetector structures have been reported, including photoelectrochemical [17], Schottky junctions [18], and p–n junctions [19]. The p–n junctions become attractive owing to their unique characteristics of no oxygen dependency and low applied fields [3]. It is known that it is hard for ZnO to achieve effective hole doping owing to several mechanisms of low solubility, compensation by low-energy native defects, and deep impurity levels [20]. Therefore, partnering suitable p-type layers with n-type ZnO has been a challenge in fabricating high-performance photodetectors. Sabina et al. fabricated a ZnO NR/CuSCN UV photodetector, which exhibited responsivity of 7.5 mA W^−1^ at −5 V and rise/decay times of 500 ns/6.7 μs, respectively [21]. Fu et al. reported a ZnO NRs/CuO UV photodetector showing responsivity of 0.272 mA W^−1^ and rise/decay times of 27/5 s, respectively [22]. Kawazoe et al. [23] successfully fabricated p-types conductive materials CuAlO_2_, based on the theory of chemical modification of the valence band, which attracted considerable attention. Through selecting suitable cationic species and crystal structures to modify the energy band structure, numerous CuMO_2_ (M = Al, Ga, In, Fe) materials would result in favorable optoelectronic properties. The prototypical delafossite CuAlO_2_ has a layered structure and linear conformation of Cu–O–Cu, which enlarges the band gap for transmitting visible light, and AlO_6_ octahedral units reduce the localization behavior. It is worth noting that the closed shell (d^10^s^0^) of cationic species has a comparable energy level with the O 2p orbitals, which can alleviate the localization behavior and avoid coloration due to intra-atomic excitation [23,24,25,26]. Owing to its wide bandgap (>3 eV) and high visible transmittance of 60–80%, CuAlO_2_ has been synthesized in various techniques including sol–gel synthesis, magnetron sputtering, and hydrothermal methods [27,28,29]. The hydrothermal method is commonly used to prepare delafossite structure oxides because of the simple procedure, easy doping, low cost, and up-scalable alternative. However, the conductivity for CuAlO_2_ is only three to four orders of magnitude lower than the n-type transparent conducting oxides (TCOs). This characteristic is attributed to poor p-type conductivity and low hole mobility, which is caused by low acceptor defects density and high acceptor ionization energy [30]. Therefore, numerous post-treatments were applied to optimize the properties of the materials. An effective way is to use various valence state dopants, such as Ag, Mg, N, and CuO, to further enhance the p-type conduction [30,31,32,33]. Dong et al. reported that the conductivity of CuAlO_2_ was enhanced by three orders of magnitude by adding Mg [34]. Yao et al. reported the doped CuAlO_2_ thin films prepared by magnetron sputtering showed high Hall mobilities of 11.3–39.5 cm^2^/Vs, and controllable p-type conductivity. Moreover, CuAlO_2_ thin films have been extensively used in various photoelectric fields such as photocatalysis [35], solar cells [36], and transistor [37]. Pr’evot et al. showed that mesoporous CuAlO_2_ was used as a scaffold to construct a CuAlO_2_/CuFeO_2_ host–guest composite electrode, which improved charge transport and decreased charge recombination [38]. Bottom-gate p-type thin-film transistors with a CuAlO_2_ channel layer were fabricated on a SiO_2_/Si substrate, which exhibited a current on/off ratio of 10^3^ and a hole mobility of 0.1 cm^2^ V^−1^ s^−1^ [37]. The CuAlO_2_/CuO was also utilized as the hole-transporting layer in inverted perovskite-based solar cells with power conversion efficiency of 16.3% [39]. The favorable electrical property originates from incorporating CuO in the CuAlO_2_ lattice to enhance the p-type conduction via the synergistic effects of energy band bowing [30,40]. The Si/CuAlO_2_ heterostructure photodetectors have been shown to exhibit high responsivity approaching 541 mA/W at 2.5 V bias voltage [41]. In spite of wide direct bandgap energy and favorable type-II band alignment with ZnO for CuAlO_2_, few photodetectors based on ZnO NRs/CuAlO_2_ heterostructures have been explored so far.

In this work, we present the hybridization of Cu^2+^ in CuAlO_2_ in the form of crystalline CuO clusters and the characterization of FTO/ZnO NRs/CuAlO_2_/Au UV photodetectors using the drop-coating method. The UV photodetectors exhibit excellent photoelectric characteristics. Also, this work provides a new pathway towards high performance UV photodetectors with the delafossite CuMO_2_ materials.

## 2. Experimental Section

### 2.1. Fabrication of ZnO/CuAlO_2_ Heterojunction

A hydrothermal method was used to prepare the ZnO NRs on fluorine-doped tin dioxide (FTO) glass substrate. In detail, the FTO glass substrate was sonicated with deionized water. ZnO precursor solution was prepared by dissolving appropriate zinc acetate dihydrate (98.0–101.0%, Alfa Aesar (China) Chemicals Co., Ltd., Shanghai, China) and 0.035 mol diethanolamine (99%, Alfa Aesar (China) Chemicals Co., Ltd., Shanghai, China) in 50 mL isopropanol (≥99.7%, Shanghai Titan Scientific Co., Ltd., Shanghai, China). After stirring evenly, the transparent precursor solution was deposited by spin-coating onto the FTO glass substrate and annealed in air atmosphere at 300 °C for an hour. Then the substrates glass covered with a seed layer was dipped into a 150 mL Teflon-lined steel autoclave, consisting of equimolar solution hexamethylenetetramine (≥99.0%, Chengdu Kelong Chemical Co., Ltd., Chengdu, China) and zinc nitrate hexahydrate (≥99.5%, Chengdu Kelong Chemical Co., Ltd., Chengdu, China). The autoclave was heated at 85 °C for four hours. Finally, the grown nanorods were annealed to obtain better crystallization properties.

The CuAlO_2_ was also fabricated via a hydrothermal process. Firstly, a 3:1 mole ratio of cuprous chloride (≥97.0%, Chengdu Kelong Chemical Co., Ltd., Chengdu, China) and sodium meta-aluminate (≥41.0%, Shanghai Titan Scientific Co., Ltd., Shanghai, China) was dissolved in 50 mL deionized water, followed by 0.6 g sodium hydroxide (≥98.0%, Chengdu Kelong Chemical Co., Ltd., Chengdu, China). After stirring for more than an hour at room temperature, the well-mixed precursor solution was sealed in a 100 mL Hastelloy autoclave. The autoclave was first heated in an oven at 400 °C for four hours, and then cooled to room temperature naturally. The precipitate was washed with diluted hydrochloric acid (36.0–38.0%, Chengdu Kelong Chemical Co., Ltd., Chengdu, China) solution (1 mol/L), diluted ammonia (25.0–28.0%, Chengdu Kelong Chemical Co., Ltd., Chengdu, China) solution (1 mol/L), deionized water, and absolute alcohol in sequence several times. After drying in air at 60 °C for five hours, the samples were stored in a low-temperature and dry environment. Meanwhile, 1.8:1 mole ratio of cuprous chloride and sodium meta-aluminate was dissolved in 50 mL deionized water, followed by 0.6 g sodium hydroxide in order to obtain alloying non-isovalent Cu–O with CuAlO_2_. Using the same synthesis steps, the precursor solution was stirred evenly and loaded into a Hastelloy autoclave. For another, the autoclave was placed into the oven at 360 °C for four hours. Also, the powder was washed in the same way.

CuAlO_2_ and CuAlO_2_/Cu–O crystals were dissolved in ethanol, which was placed in an ultrasonic for more than four hours to obtain stable and uniform dispersion. This dispersion was dropped on the preheated ZnO NRs and annealed in air at 300 °C for 20 min. Au was evaporated as an anode via electron beam evaporation. Finally, the device was scribed to an area of 0.09 cm^2^ to form a vertical structure of FTO/ZnO NRs/CuAlO_2_ (or [CuAlO_2_/Cu–O])/Au.

### 2.2. Characterization

The structure of prepared samples was measured using X-ray diffraction ( XRD-6100, Shimazu, Tokyo, Japan) using Cu Ka radiation (λ = 1.5406 Å, 30 kV and 10 mA). The material morphologies were characterized by the scanning electron microscopy (SEM, ZEISS Gemini 300, ZEISS, Oberkochen, Germany) equipped with an X-ray energy-dispersive spectrometer (EDS). (The acceleration voltage is 3 kV when the topography is photographed, and the acceleration voltage is 15 kV when the energy spectrum mapping is photographed. The probe beam is 3 pA-20 nA, and the stability is better than 0.2%/h.) X-ray photoelectron spectroscopy (XPS, Thermo Scientific K-Alpha, Thermo Fisher Scientific Inc., Waltham, MA, USA) was used to investigate the chemical state of Cu element, which were calibrated based on C 1s peak 284.8 eV (The spot size is 400 μm, the working voltage is 12 kV, and the filament current is 6 mA. The full-spectrum scanning energy is 150 eV, and the step size is 1 eV. The narrow-band scanning pass energy is 50 eV, and the step size is 0.1 eV). The work functions of the samples were measured by the ultraviolet photoelectron spectroscopy (UPS, Thermo ESCALAB XI+, Thermo Fisher Scientific Inc., Waltham, MA, USA). The optical properties of the sample were conducted in the wavelength range from 250 to 800 nm with PerkinElmer Lambda 950 UV–visible spectrometer (PE 950, PerkinElmer Inc., Waltham, MA, USA). From the manufacturer’s data analysis, the measurement error associated with the transmission value is estimated to be about 0.25%. Electrochemical impedance spectra (EIS) were carried out on electrochemical workstation (CHI660E, CH Instruments, Inc., Shanghai, China) under dark conditions in the frequency range from 10^6^ to 10^−1^ Hz. The external and apparent quantum efficiency (EQE and AQE, respectively) in the range of 320–440 nm was measured by QEX10 (PV Measurements, Inc, Washington, USA) at room temperature. It was calibrated with a standard Si solar cell before measuring. The light source was a xenon lamp with a power of 65 mW cm^−2^ and the frequency of the chopper was 120 Hz. The light was incident from the glass surface, and the spot diameter on the sample was 2 mm. The current–voltage (I–V) and time-resolved current curves were collected through Keithley 2400 digital source meter. The effective illumination area of the device was 3.14 mm^2^.

## 3. Results and Discussion

**Figure 1:**
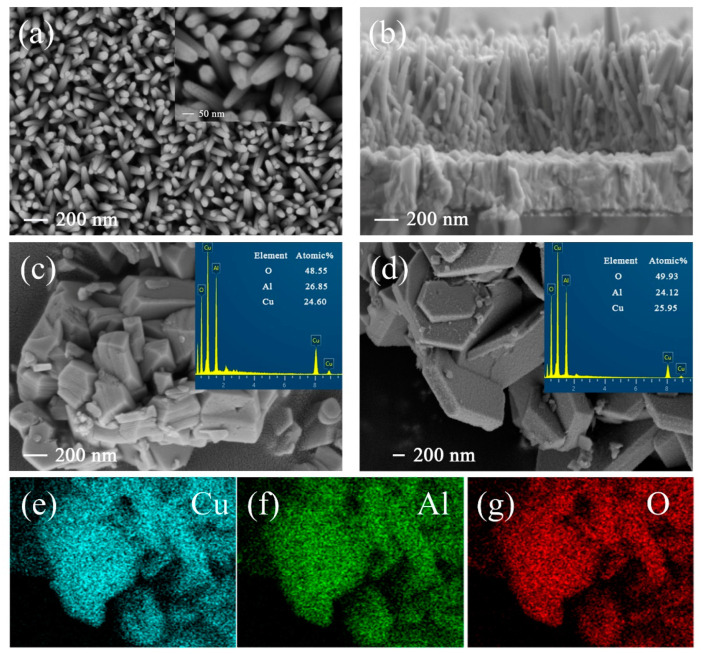
(**a**) Top-view and (**b**) cross-sectional view SEM images of ZnO NR arrays; top-view SEM images of (**c**) pure CuAlO_2_ and (**d**) [CuAlO_2_/Cu–O]; elemental mapping of (**e**) Cu, (**f**) Al, and (**g**) O of [CuAlO_2_/Cu–O].

Figure 1 displays the morphological analysis of ZnO NRs, CuAlO_2_, and typical CuAlO_2_/Cu–O powders. From the top-view and cross-sectional SEM images of ZnO NRs, one can see that vertically grown nanorod arrays with a diameter of ~50 nm (Figure 1a) and an average thickness of about 850 nm distribute uniformly on the FTO substrates (Figure 1b). This optical trapping structure of nanorods can improve the absorption of ultraviolet light [42]. Figure 1c shows the SEM and EDS results of the pure CuAlO_2_ sample, which is uniform, compact, and smooth with particle sizes of 200–400 nm. This polygonal grain shape suggests that the treatment process, such as sonication, does not alter its crystallinity [39]. The illustration in Figure 1c reveals that the elemental percentages of Cu (~24.60 at.%), Al (~26.85 at.%), and O (~48.55 at.%) match well with the stoichiometric proportion of pure CuAlO_2_. In Figure 1d, Cu–O incorporation into CuAlO_2_ obviously enhances the grain size (400–600 nm). The CuAlO_2_/Cu–O is rough and flaky with randomly grown minor crystallites. This flaky particle is beneficial for the improvement of the conductivity of CuAlO_2_, as reported by other researchers [43]. It can be found that the atomic ratio of Al decreases with the incorporation of Cu–O clusters. Elemental mapping of CuAlO_2_/Cu–O (Figure 1e–g) reveals that the film is mainly composed of Cu, Al, and O, which distribute homogeneously in the sample.

**Figure 2:**
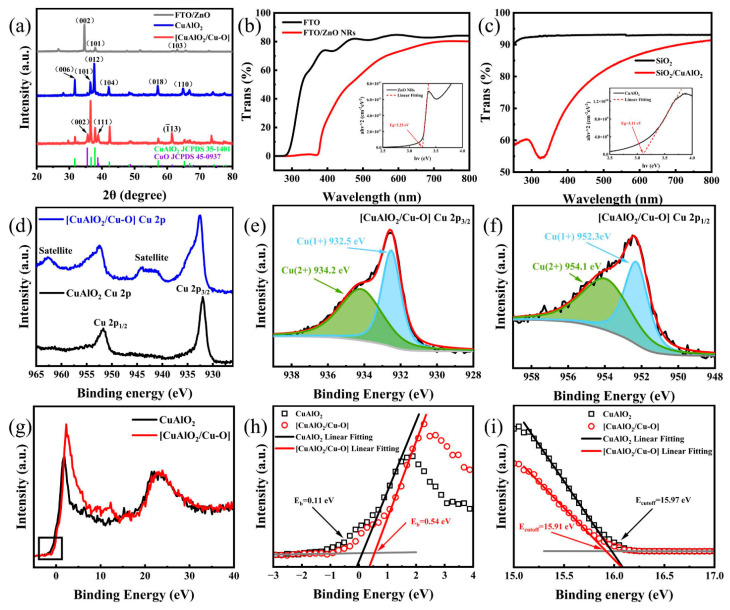
(**a**) XRD patterns of ZnO, CuAlO_2_, and [CuAlO_2_/Cu–O], (**b**) UV–vis spectra of FTO and FTO/ZnO NRs (inset showing the Tauc plot of ZnO NRs), (**c**) UV–vis spectra of SiO_2_ and SiO_2_/CuAlO_2_ (inset showing the Tauc plot of CuAlO_2_), (**d**) Cu 2p XPS spectra of CuAlO_2_ and [CuAlO_2_/Cu–O], (**e**) Cu 2p_3/2_ and (**f**) Cu 2p_1/2_ of [CuAlO_2_/Cu–O], (**g**) XPS wide valence-band spectra of CuAlO_2_ and [CuAlO_2_/Cu–O] and (**h**) their particular XPS wide valence-band spectra, (**i**) UPS spectra of CuAlO_2_ and [CuAlO_2_/Cu–O].

Figure 2a shows the XRD pattern of ZnO nanorods, which is consistent with the hexagonal wurtzite ZnO (JCPDS card 36-1451) [44]. The (002) peak with the strongest intensity is located at 34.6°, indicating that the ZnO NRs have high c-axis growth orientation. It is observed that other weak peaks at 43.7° and 60.1° have the orientations along (102) and (103), respectively. There are no impurity peaks observed in the ZnO NRs. Also in Figure 2a, the peaks center at 31.6°, 36.6°, 37.8°, 42.2°, and 57.2°, corresponding to (006), (101), (012), (104), and (018), respectively, of CuAlO_2_ (JCPDS card 35-1401) [28]. As for CuAlO_2_/Cu–O, there are extra characteristic peaks 35.4°, 38.7°, and 61.5°, representing the (002), (111), and ($\bar{1}$13), respectively, which are ascribed to CuO compounds (JCPDS card 45-0937) [45]. 

The optical transmittance for ZnO NRs and CuAlO_2_ films was examined by using UV–vis spectroscopy from 250 to 800 nm, as shown in Figure 2b,c. The illustration of Figure 2b,c shows the (ahν)^2^ versus hν of ZnO NRs and CuAlO_2_ films. CuAlO_2_ has more than 70% transmittance in the visible region and a significant absorption at about 340 nm. The energy band gap of CuAlO_2_ films (~3.11 eV) can be determined using the Tauc’s formular [46]. The organic residues or high grain boundary scattering can lead to significant optical absorption below the bandgap energy [47]. ZnO has more than 60% transmission in the visible region, and ZnO NRs can mainly absorb UV light. The calculated band gap for ZnO NRs is about 3.25 eV, which is lower than that for bulk ZnO (~3.37 eV) at room temperature. It is because the defect density increases with the increase in specific surface area, which leads to the formation of defect levels in the band gap of ZnO [6].

To verify the hybridization of Cu–O dimers with CuAlO_2_ host, XPS was used to investigate the chemical state of the compound, which witnesses the variation in Cu valence states (Figure 2d–f). The spectrum of CuAlO_2_ (Figure 2d) manifests that the Cu 2p_3/2_ and Cu 2p_1/2_ related peaks are found at 932.0 and 951.7 eV, respectively, which is consistent with prior work [48,49,50]. The monovalent state of copper (Cu^+^) can be confirmed through this phenomenon, as there is no shake-up line of Cu 2p_3/2_ in the spectrum. After the hybridization of Cu–O dimers (Figure 2e,f), the binding energies of Cu 2p_3/2_ and Cu 2p_1/2_ of CuAlO_2_ slightly shift to 932.5 eV and 952.3 eV, respectively [28]. This is because the strong two-dimensional confinement of Cu–O bonds restrains the electronic structure [51]. The two weak peaks appear at 934.2 eV and 954.1 eV, corresponding to the binding energies of Cu 2p_3/2_ and Cu 2p_1/2_, respectively, for CuO [52]. Furthermore, the difference between these two energy levels is close to 20 eV and the core level spectrum of Cu^2+^ compounds has a strong satellite feature (shake-up satellite) caused by 3d vacancy states in the electronic configurations of atoms [53,54]. This feature has been widely used to distinguish Cu^+^ from Cu^2+^ in various copper compounds. Quantitative analysis of the XPS spectra provides Cu^2+^/Cu^+^ atomic ratios of 1.2 for CuAlO_2_/Cu–O. XPS and XRD measurements indicate that Cu–O is hybridized with the CuAlO_2_ lattice in the forms of crystalline CuO clusters, which is also in agreement with previously reported results [30]. From the XPS valence band spectra in Figure 2g,h, the valence band positions of CuAlO_2_ and CuAlO_2_/Cu–O are 0.11 eV and 0.54 eV lower than Fermi level, respectively. 

Next, the work function of CuAlO_2_ and CuAlO_2_/Cu–O was investigated from the UPS measurements (Figure 2i). The work function can be estimated from this equation [55]:

(1) $$\Phi =hv-\left(E_{cutoff}-E_{fermi}\right)$$

Through a linear extrapolation of the secondary electron cut off edge, the *E_cutoff_* of CuAlO_2_ is about 15.97 eV in the high binding energy. Combined with the energy of the incident photon, the work function for CuAlO_2_ is calculated to be about 5.25 eV, while that for CuAlO_2_/Cu–O is about 5.31 eV. In addition, the ionization potential increases from 5.36 to 5.85 eV. These results manifest that the valence band maximum (VBM) keeps away from the vacuum level and the binding energy increases through the hybridization of Cu–O with the CuAlO_2_ host. CuAlO_2_/Cu–O is not the physical mixture of CuO clusters in the CuAlO_2_ matrix. CuO clusters can induce the formation of Cu^2+^ 3d^9^ orbitals, which are easier to activate in the VBM of CuAlO_2_ [56,57], thus, enhancing the p-type conduction of CuAlO_2_ [30]. Hence, the energy band level and electronic properties of CuAlO_2_ can be adjusted by alloying Cu–O with a CuAlO_2_ host. Other Cu^+^-based ternary p-type transparent oxides also experience a similar valence band shift effect after optimizing their electrical properties [24].

**Figure 3:**
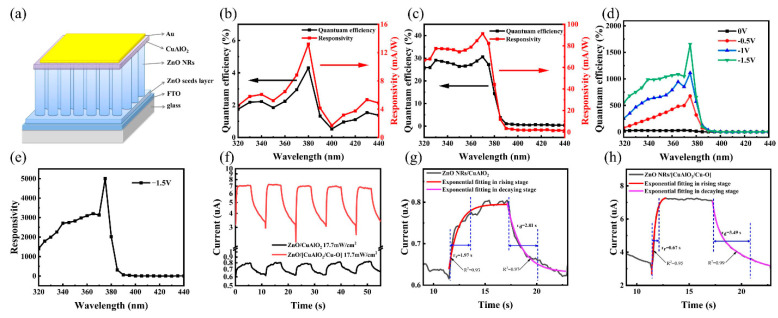
(**a**) Stack of the self-powered UV photodetector; EQE and responsivity of (**b**) ZnO/CuAlO_2_ and (**c**) ZnO/[CuAlO_2_/Cu–O] device at 0 V; (**d**) EQE of ZnO/[CuAlO_2_/Cu–O] device at different bias voltages; (**e**) responsivity of ZnO/[CuAlO_2_/Cu–O] device with 1.5 V bias; (**f**) time-dependent photoresponse of the ZnO/CuAlO_2_ and ZnO/[CuAlO_2_/Cu–O] devices; the rise and decay time fitting in a single on–off circle of (**g**) ZnO/CuAlO_2_ and (**h**) ZnO/[CuAlO_2_/Cu–O] devices.

To demonstrate the application of the delafossite materials, ZnO-NRs-based photodetectors were fabricated in this work (Figure 3a). Figure 3b,c reveal the external quantum efficiency (*EQE*) of the as-fabricated ZnO/CuAlO_2_ and ZnO/[CuAlO_2_/Cu–O] UV detectors at corresponding wavelengths of the UV light at zero bias. The performance of the UV photodetector is typically characterized by the responsivity (*R*), which can be calculated by using the following equation [58,59]:

(2) $$R_{\lambda }=\frac{q\lambda EQE}{hc}\times 100\%$$

where *λ* is the radiation wavelength, *c* is the speed of light, and h is the Planck constant. The response wavelength range is from 320 to 440 nm and the peak responsivity is found at 370–380 nm, which correlates well with the high optical absorptance lower than 400 nm wavelength. The device is visible–blind ultraviolet detection, which is consistent with the results of transmittance spectra. The peak responsivity of the ZnO/[CuAlO_2_/Cu–O] photodetector is about 91.3 mA/W, higher than the 13.2 mA/W of ZnO/CuAlO_2_ photodetector, indicating that the incorporation of Cu^2+^ significantly improves the performance of the device. It is ascribed to the increase in the photocurrent, as more carriers are collected under illumination after the incorporation of Cu^2+^ in the CuAlO_2_ thin films. The maximum responsivity reveals the high capability to detect UV signals and the presence of internal gain in the device. The related UV-to-visible rejection ratio (375–440 nm) of the devices is about 75, which confirms the UV spectrum selectivity. This is attributed to the wide bandgap of the ZnO (~3.25 eV) and the CuAlO_2_ (~3.11 eV) semiconductors, which leads to a dramatic increase in UV light absorption in the active material of the device. There is no significant change in the quantum efficiency for wavelengths higher than 400 nm, which may be related to the reduction in photogenerated carrier due to the decrease in the optical absorptance of ZnO [47]. After applied bias voltage, the photoresponsivity for the photodetector increases up to 5002 mA/W at −1.5 V under 375 nm wavelength (Figure 3d,e). This enhancement is attributed to the fact that applying a negative bias can enhance the width of built-in electric field, which leads to separate photogenerated carriers more effectively. In short, the ZnO/[CuAlO_2_/Cu–O] detectors deliver the high performance in the UV–A region.

Furthermore, the specific detectivity is used to characterize the sensitivity of the photodetector. The higher the specific detectivity, the better the detection ability of the device for weak signals. It can be calculated using the following formula [47]:

(3) $$D^{*}=\frac{R}{\sqrt{2qJ_{dark}}}$$

where *D*^∗^ is the specific detectivity and *J_dark_* is the dark current density at bias voltage. The detectivity of ZnO/CuAlO_2_ is calculated to be 2.35 × 10^10^ cm Hz^1/2^ W^−1^. The incorporation of Cu–O clusters enhances the hole transport capacity and increases the photocurrent density, thereby improving the responsivity. Therefore, the ZnO/[CuAlO_2_/Cu–O] detector shows a higher detectivity of 1.71 × 10^11^ cm Hz^1/2^ W^−1^ for weak signal.

Figure 3f shows the stability and the time response characteristics of the devices measured at −1 μV bias under illumination of 17.7 mW/cm^2^ at 365 nm. All the devices demonstrate excellent stability and repeatability in five cycles. The photogenerated current of the ZnO/[CuAlO_2_/Cu–O] device is about 6.99 μA, significantly higher than the ZnO/CuAlO_2_ device (~0.78 μA). The photocurrent enhancement is mainly due to the reduction in carrier recombination by incorporating Cu–O clusters, which is in agreement with the previous study [39]. Under UV illumination, ZnO NRs absorb photons to generate electron–hole pairs, and holes migrate toward the ZnO surface, discharging the negatively charged adsorbed oxygen ions (O_2(ad)_^−^ + h^+^ → O_2(g)_). After the UV light is off, the photocurrent decay is governed by the slow surface-related process. Oxygen molecules adsorbed physically on the ZnO surface capture electrons from the conduction band to become chemisorbed oxygen (O_2(g)_ + e^−^ → O_2(ad)_^−^) [12,60,61]. Therefore, the response processes can be fitted by the following biexponential decay model [62]:

(4) $$I_{t}=I_{0}+A_{1}e^{\left(-\frac{t}{\tau_{1}}\right)}+A_{2}e^{\left(-\frac{t}{\tau_{2}}\right)}$$

where τ_1_ and τ_2_ are two relaxation time constants. The rise time is defined as the time to rise from 10% to 90% of the highest photocurrent value after UV illumination, and decay time is defined as the time to fall from 90% to 10% of the highest photocurrent value after switching off UV illumination. More technically, R^2^ is a measure of goodness of fit, and its value ranges from 0 to 1. When the R-squared value of the trend line is closer to 1, the degree of fitting is better and the trend line is much more reliable. The rise and decay times of the ZnO/CuAlO_2_ detector are 1.97 s and 2.81 s in Figure 3g, respectively. The rise and fall times of the ZnO/[CuAlO_2_/Cu–O] detector are 0.67 s and 3.49 s in Figure 3h, respectively. The hybridization of Cu–O does not significantly improve the ZnO NRs/CuAlO_2_ interface, so it cannot effectively improve the response time. In ZnO-NRs-based photodetectors, the response time is relatively long due to the slow rate of oxygen adsorption and desorption on the surface of ZnO NRs.

**Figure 4:**
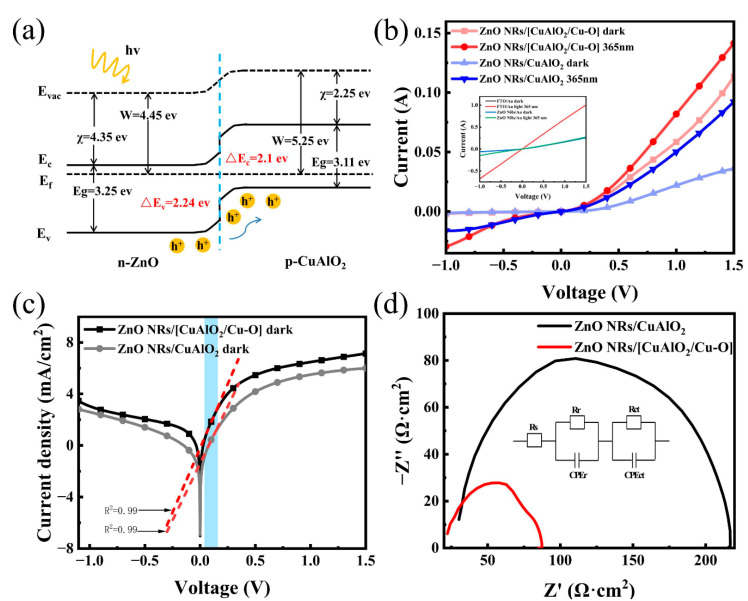
(**a**) Energy band diagram of the ZnO NRs/CuAO_2_ heterojunction, (**b**) I–V curves of ZnO NRs/CuAlO_2_ and ZnO NRs/[CuAlO_2_/Cu–O] devices in the dark and upon 365 nm light (I–V curves of FTO/Au and FTO/ZnO NRs/Au inside), (**c**) the plot of lnJ vs. V in the dark, (**d**) EIS spectra of the ZnO NRs/CuAlO_2_ and ZnO NRs/[CuAlO_2_/Cu–O] devices.

To help understand the carrier transport process, the schematic energy band diagram of the UV photodetector based on the ZnO/CuAlO_2_ heterojunction is shown in Figure 4a. The bandgap of ZnO is about 3.25 eV, while the electron affinity and work function have been reported to be about 4.35 and 4.45 eV, respectively[63]. The values of the band gap, the work function, and valence band position for CuAlO_2_ are 3.11, 5.25, and 5.36 eV, respectively, in this work. The type II band alignment between ZnO and CuAlO_2_ facilitates carrier separation and transportation. When UV light is irradiated from FTO glass, ZnO absorbs photons to generate electron–hole pairs predominantly. Under the action of the built-in electric field, the electron–hole pairs are separated and collected into the electrode, resulting in the formation of the photocurrent. The CuAlO_2_, as the p-type layer, is beneficial for carrier separation. The reason is that CuAlO_2_ as an energy barrier layer reduces the recombination rate, and improves the electron injection into the ZnO layer [29,64].

Figure 4b,c shows the I–V characteristics of the ZnO/CuAlO_2_ heterostructure photodetector in the dark and under 365 nm UV illumination. The I–V curves of the FTO/Au and FTO/ZnO NRs/Au devices are also in the illustration of Figure 4b. The photocurrent curve coincides well with the dark current curve, indicating that the FTO and Au electrode are ohmic contacts. As for the ZnO NRs/Au device, it shows a relatively weak photoresponse. However, the obvious rectifying diode characteristic for ZnO/CuAlO_2_ confirms the formation of a p–n junction rather than a Schottky junction. The ZnO/CuAlO_2_ heterojunction with the forward current density to the reverse current density ratio is 18 at ±1 V, and is lower than the ratio of 30 at ±1 V for the diode made of ZnO/[CuAlO_2_/Cu–O] (Figure 4b). It demonstrates the ability of ZnO/CuAlO_2_ heterojunctions to separate photogenerated carriers. Moreover, the incorporation of Cu^2+^ can also improve the electrical conductivity. The current increases greatly when the ZnO/[CuAlO_2_/Cu–O] heterostructure photodetector is illuminated by UV light compared with dark current at −1 V, indicating a highly UV-sensitive photoconduction and photocurrent. The strength of the space electric field is increased with the increase in reverse bias, resulting in a more efficient separation of the photogenerated carriers. Therefore, the ZnO/[CuAlO_2_/Cu–O] heterojunction can further enhance the electrical properties.

In addition, the diode ideality factor (*n*) and dark saturation current density (*J*_0_) can be extracted from semi-log plot of the I–V curve using the following Shockley ideal diode equation [65,66]:

(5) $$J=J_{0}[exp(\frac{qv}{nkT})-1]$$

where *J* is the dark current density, *J*_0_ is the reverse current density, *v* is the applied voltage, *n* is the ideality factor, *T* is the absolute temperature, and *k* is the Boltzmann constant. In order to compare the p–n junction characteristics of the two devices, we selected the same linear region (the blue line) to fit the LnJ–V curves. The shape of I–V curves depends on the height of the Schottky barriers at the interface of semiconductors. As for the UV photodetectors, the *n* drops from 2.3 to 1.9 and *J*_0_ decreases from 0.81 to 0.24 mA cm^−2^. The relatively large *J*_0_ can be attributed to the presence of structure imperfections and high density of interface traps in the ZnO/CuAlO_2_ heterojunction, causing a current tunnel path and increasing the reverse current [67]. Thus, through the incorporation of Cu^2+^ in CuAlO_2_, high-quality CuAlO_2_/Cu–O films and a high-performance heterojunction were achieved.

To gain more insights into the reason for device performance improvement, the EIS measurements were used to investigate interfacial carrier separation and transfer ability in the frequency range from 0.1 Hz to 1 MHz in the dark (Figure 4d). From Figure 4d, one can see that the resistances consist of charge transfer resistance (R_ct_) obtained in the high-frequency region, recombination resistance (R_r_) obtained in the low-frequency region, and series resistance (R_s_) originating from a combination of contact resistance in the overall circuit and electrolyte [68]. The constant phase element (CPE) is the non-ideal frequency-dependent capacitor. By the analysis of Zview, the ZnO/[CuAlO_2_/Cu–O] detector has a much lower charge transfer resistance (~27.84 Ω) than that of the ZnO/CuAlO_2_ (~121.2 Ω). The results suggest that the incorporation of Cu^2+^ leads to a more effective charge separation and a faster interfacial charge transfer.

Table 1 lists several parameters of self-powered ZnO NRs/[CuAlO_2_/Cu–O] photodetectors and other ZnO-based p–n junction UV detectors. We can see that the responsivity of ZnO/[CuAlO_2_/Cu–O] is lower than the n-ZnO/CdS/p-GaN structure under zero bias. The excellent responsivity of the n-ZnO/CdS/p-GaN structure is due to the CdS insert layer, which is used as a carrier transition layer to effectively reduce the interfacial charge recombination, resulting in better photoresponse characteristics. ZnO NRs/[CuAlO_2_/Cu–O] has a simple structure, which is through hybridizing Cu–O clusters into the CuAlO_2_ lattice to adjust the energy band and enhance the conductivity of the materials, thereby improving the performance of photodetector. It has a higher responsivity of 5.002 A/W at 1.5 V. Compared with other ZnO-NRs-based photodetectors, ZnO NRs/[CuAlO_2_/Cu–O] shows higher response and excellent detectivity, indicating that CuAlO_2_/Cu–O is a promising p-type material for optoelectronic community. 

## 4. Conclusions

In summary, a novel ZnO-NRs-based UV photodetector was prepared by all-wet synthesis methods at relatively low temperatures. The photodetector has a good self-powered performance due to good type II band alignment. Further, hybridizing Cu–O clusters into the CuAlO_2_ lattice can adjust the energy band and enhance the conductivity of the materials, thereby improving performance of photodetector. The ZnO/[CuAlO_2_/Cu–O] photodetector delivers a strong responsivity of 91.3 mA/W at 0 V bias, which is almost seven times higher than the ZnO/CuAlO_2_ photodetector (~13.2 mA/W) under UV radiation of 65 mW cm^−2^. The detectivity also increases from 2.35 × 10^10^ to 1.71 × 10^11^ Jones. Particularly, at 1.5 V bias, the responsivity for the ZnO/[CuAlO_2_/Cu–O] photodetector is about 5002 mA/W under 375 nm UV illumination. Therefore, hybridizing Cu–O clusters provides a new idea to improve the electrical properties of the delafossite CuMO_2_ (M = Al, Ga, In, Fe) materials for high-performance optoelectronic devices.

## Figures and Tables

**Table 1:** Comparison of photoresponse parameters and test conditions between ZnO-based p–n junction photodetectors reported and this work.

Heterostructure	Wavelength	Rectification Ratio	Responsivity (Bias Voltage)	Rise Time Decay Time	Detectivity (Jones)	Ref.
Cl-ZnO NRs/DMSO-PEDOT: PSS	365 nm	-	0.8 mA W^−1^ (0 V)	0.03 s 0.032 ms	1.12 × 10^10^	[69]
ZnO NRs/CdS/GaN	300 nm	-	176 mA W^−1^ (0 V)	<0.35 s	2.5 × 10^12^	[70]
ZnO NRs/PVK/Cu_2_O	360 nm	-	13.28 A W^−1^ (−0.1 V)	8.7 s 128.3 s	1.03 × 10^13^	[71]
ZnO NR/CuSCN	355 nm	23 (±4 V)	7.5 mA W^−1^ (0 V)	500 ns 6.7 us	-	[21]
ZnO NRs/Cu_2_O	390–480 nm	19.1 (±2 V)	60–70 mA W^−1^ (0 V)	75 ms 70 ms	-	[72]
ZnO NRs/CuO	365 nm	-	0.272 mA W^−1^ (0 V)	27 s 5 s	-	[22]
ZnO NRs/CuO/PEDOT:PSS	365 nm	-	9.96 mA W^−1^ (0 V)	33 ms 296 ms	-	[73]
ZnO NRs/CuSCN/rGO	375 nm	5690 (±1 V)	18.65 mA W^−1^ (−1 μV)	105 ms 100 ms	3.8 × 10^11^	[74]
ZnO NRs/CuI	380 nm	17.7 (±1 V)	86.84 mA W^−1^ (0 V)	110 ms 110 ms		[66]
ZnO NRs/CuAlO_2_	380 nm	18 (±1 V)	13.2 mA W^−1^ (0 V)	1.97 s 2.81 s	2.35 × 10^10^	This work
ZnO NRs/[CuAlO_2_/Cu–O]	370 nm	30 (±1 V)	91.3 mA W^−1^ (0 V)	0.67 s 3.49 s	1.71 × 10^11^	This work
	375 nm	-	5.002 A W^−1^ (−1.5 V)	-	-	This work

## Data Availability

The data that support the findings of this study are available from the corresponding author upon reasonable request.
